# Adverse events following immunisation (AEFI) reports from the Zimbabwe expanded programme on immunisation (ZEPI): an analysis of spontaneous reports in Vigibase® from 1997 to 2017

**DOI:** 10.1186/s12889-019-7482-x

**Published:** 2019-08-27

**Authors:** Josiah Tatenda Masuka, Star Khoza

**Affiliations:** 1 Harare Central Hospital, PO Box ST14, Southerton, Harare, Zimbabwe; 2 Department of Dermatology, Nelson R Mandela School of Medicine, Private Bag X7, Congella, Durban, 4013 South Africa; 3 Department of Clinical Pharmacology, College of Health Sciences, University of Zimbabwe, PO Box A178, Avondale, Harare, Zimbabwe; 4 Discipline of Pharmacology and Clinical Pharmacy, School of Pharmacy, Faculty of Natural Sciences, University of the Western Cape, Private Bag X17, Bellville, 7535 South Africa

**Keywords:** AEFI reporting, Vaccine safety, Pharmacovigilance, Immunization, Children

## Abstract

**Background:**

Vaccine safety surveillance is an essential requirement in vaccination programmes. It supports signal identification, hypothesis generation, and the identification and rectification of gaps in vaccine pharmacovigilance systems. The objectives of this study were to determine the characteristics and trends of adverse events following immunisation (AEFI) and to assess the performance of the Zimbabwe Expanded Immunisation Programme safety surveillance system.

**Methods:**

We carried out a descriptive study of passively collected vaccine-related Individual Case Safety Report (ICSR) data submitted to the World Health Organization global adverse drug reaction database (VigiBase®) from Zimbabwe during the period 1997 to 2017. We extracted AEFI/ICSR data using VigiLyze® for analysis with respect to the demographic distribution, AEFI characteristics, reporting trends over time, ICSR timeliness and case completeness.

**Results:**

A total of 272 vaccine-related ICSRs were included in the analyses with a median completeness score of 0.90 interquartile range, IQR (0.63; 0.90). The overall annual reporting rate was 0.58 per 100,000 vaccine doses and the AEFI reporting ratio ranged between 0 and 30.2 AEFI reports per 100,000 surviving infants. The majority of ICSRs were male (55.3%; *p value = 0.641*) and the median age was 12 (0–168) months. The majority of ICSRs were reported in children who had received measles (*n* = 133; 48.9%) and OPV/DTP-Hib-HepB (*n* = 107; 39.3%) vaccines. Of the 387 observed AEFIs, 301 (77.8%) were systemic events and 86 (22.2%) were local reactions. Systemic events were more frequently reported with doses containing the measles antigen (*n* = 190; 49.1%) while local events were associated with the multiple antigen OPV/DTP-Hib-HepB (*n* = 62; 16.0%). The multiple antigen OPV/DTP-Hib-HepB was associated with higher rates for injection site abscess (*n* = 57), pyrexia (*n* = 27), diarrhea (*n* = 15), vomiting (*n* = 12), and seizures (*n* = 6). The measles antigen was associated with higher rates for rash (*n* = 44), ocular disorders (*n* = 26), pyrexia (*n* = 26), urticaria (*n* = 22), diarrhea (*n* = 8), and vomiting (*n* = 12).

**Conclusions:**

Most of the ICSRs were associated with measles and OPV/DTP-Hib-HepB vaccines. Zimbabwe’s vaccine safety surveillance system is still developing and is not yet fully functional. However, the current system provides a reference point for the monitoring of the ongoing AEFI reporting trends and characteristics.

## Background

The World Health Organisation (WHO) mandates the systematic collection, analysis and evaluation of medically important adverse events following immunisation (AEFI) for all immunisation programmes [1, 2]. The major goal of this immunisation safety surveillance is the “early detection and analysis of adverse events to allow for appropriate and quick responses to emerging AEFI issues in order to decrease the negative impact on the health of individuals and the immunisation programme” [1, 3]. In addition, vaccine safety surveillance allows signal identification, hypothesis generation, and the identification and rectification of gaps in the system to strengthen the Expanded Programme on Immunisation (EPI programme) [1, 4].

Careful and continuous analysis of the post-marketing vaccine safety surveillance data provides a means to critically evaluate and communicate up-to-date information to the public on the benefit-risk profiles of individual vaccines. This helps to counter the negative perceptions on vaccination and the resultant vaccine hesitancy by improving the transparency in the immunisation programmes [5, 6]. A good example is provided by the Australia surveillance system which collates and reviews AEFI data submitted to national medicines regulator, the Therapeutic Goods Administration (TGA) annually since 2003 [7]. Through this system, Australia regularly updates its immunisation recommendations accordingly, thereby maximising the benefit-risk balance for the registered vaccines. However, in most developing countries there is limited vaccine pharmacovigilance infrastructure which subsequently reduces the capacity for continuous review of AEFI data [8].

The attendant introduction of new vaccines developed specifically for tropical diseases such as dengue fever, malaria and group A meningococcal meningitis necessitates a strengthening of the AEFI surveillance systems in these regions [3, 9]. In addition, there is limited information on the performance, quality, responses to serious AEFI issues and the characteristics of the reported AEFIs in developing countries. The aim of the present study was to provide an initial assessment of the performance and quality of Zimbabwe’s AEFI surveillance system. The study provides a detailed analysis of the characteristics of the reported AEFIs and individual case safety reports (ICSRs): the demographic distribution, seriousness, timeliness, completeness, types and/or classification of the AEFIs, and the reporting trends over time. Furthermore, we explored whether there were any annual AEFI reporting trends during the study period.

## Methods

### Study setting

Zimbabwe has been a full member of the WHO Programme for International Drug Monitoring (PIDM) since 1998. Full membership to the WHO PIDM requires a demonstration of technical competence in managing ICSRs by submitting at least 20 reports to the WHO global ICSR database, VigiBase®. ADR reporting activities are coordinated through the Medicines Control Authority of Zimbabwe (MCAZ). MCAZ submits AEFI ICSRs collected from the national EPI vaccine safety surveillance programme to VigiBase® as provided in the national AEFI guidelines [10]. This system is designed to capture AEFI reports from the Zimbabwean birth cohort with an estimated official vaccine coverage of around 90% for the surviving infants (using the 2017 GAVI estimates for DTP coverage as a proxy for the national immunisation coverage) [11]. The country has a relatively strong immunisation programme as indicated by a less than 10% drop-out rate between DTP1 and DTP3 coverage [12].

AEFI surveillance and investigation is managed and coordinated by the Zimbabwean Expanded Programme of Immunisation (ZEPI) team, a division of the Ministry of Health and Child Care. All the collected AEFI ICSRs are sent to the Medicines Control Authority of Zimbabwe for further analysis, causality assessment and subsequent upload into VigiBase®. The causality assessment is done using the WHO Aide Memoire tool by the national (central) expert committee which comprises of paediatricians, physicians, pharmacists, clinical pharmacologists and a vaccination nurse. Further investigations and information is provided by the ZEPI team when needed by the expert committee to finalise AEFI causality assessments [10].

### Study design

We carried out a descriptive study using passively collected spontaneous AEFI surveillance data from Zimbabwe covering the period 1997 to 2017. The de-identified Individual Case Safety Reports (ICSRs) were extracted from VigiBase® using its search and analysis software database known as VigiLyze® on 2017-09-16, dataset date: 2017-09-10. ICSRs which met the following criteria were included in the analysis: an AEFI, at least one suspected vaccine included in the routine EPI, and an identifiable patient in accordance with the International Conference on Harmonisation (ICH) E2D guideline [13]. We excluded AEFI reports emanating from vaccines not used in the ZEPI programme. These included AEFI reports for adults (i.e. > 18 years), rabies and tetanus vaccines.

An AEFI was defined as any untoward medical occurrence which follows immunisation and which does not necessarily have a causal relationship with the usage of the vaccine [1]. The denominator for calculating the AEFI rates was derived by multiplying the population of children immunized by the estimated coverage. The population and vaccination coverage estimates used were obtained from the 2017 GAVI Zimbabwe country factsheet [11]. Based on the 2017 GAVI Zimbabwe country factsheet, we assumed a birth cohort of 537,364 children, 515,965 surviving infants, 90% vaccination coverage, and 20 birth cohorts from 1997 to 2017 to calculate the AEFI reporting rates (per 100,000 vaccine doses). Assuming that 464,368 children of each birth cohort are vaccinated, a total population of 9,287,360 children was used as the denominator to estimate the administered vaccine doses. In cases of co-administration of two or more vaccines in an individual, we attributed the reported AEFI to the reporter suspected vaccine. The AEFI reporting ratio (per 100,000 surviving infants) as defined in the WHO’s Global Vaccine Action Plan (GVAP) for vaccine safety monitoring was calculated as a product of coverage X population using the WHO annual estimates for surviving infants from 1997 to 2017 [14]. The number of surviving infants was obtained from the United Nations Population Division statistics and the Zimbabwe GAVI country factsheet 2017 [11, 12].

Two vaccination schedules were used in the period under review differentiated by the immunisation schedule change carried out in 2012. The vaccination schedule prior to the 2012 schedule change was as follows: at birth – BCG, at 3, 4, 5 months – OPV, DTP-Hib-HepB plus/minus pneumococcal conjugate and rotavirus vaccines; at 9 months – measles; at 18 months – OPV and DTP boosters and at 5 years OPV and DTP boosters [15]. The new schedule maintains BCG at birth, measles antigen at 9 months and the OPV and DTP boosters at 18 months, but provides for administration of OPV, DTP-Hib-HepB plus/minus pneumococcal conjugate and rotavirus vaccines at 6, 10 and 14 weeks whilst dropping the booster doses previously given at 5 years of age. Table 1 shows the vaccination schedules used in Zimbabwe before 2012 and after 2012.

**Table 1:** Vaccines used for routine mandatory vaccination of children in Zimbabwe

Vaccine	Vaccination schedule before 2012 (age in months)	Vaccination schedule from 2012 (age in months)	Antigen (s)
BCG	0	0	bacillus Calmette-Guerrin
Measles	9	9	measles, rubella
OPV	3, 4, 5	6,10,14 weeks	polio
DTP-Hib-HepB	3, 4, 5	6,10,14 weeks	diphtheria, tetanus, whole cell pertussis, hepatitis b, haemophilus influenza
Pneumococcal conjugate		6,10,14 weeks	pneumococcal conjugate
OPV booster	18, 60	18	polio
DTP booster	18, 60	18	diphtheria, tetanus, whole cell pertussis

### Statistical analysis

The de-duplicated, MedDRA version 20.0 coded data was exported onto a Microsoft Office Excel™ package (Microsoft Corporation, Redmond, WA, USA). Further analysis was done using the Statistical Package for Social Sciences (SPSS) version 22.0 (IBM Corporation, Somers, NY, USA) and Stata Version 12 (College Station, TX, USA).

Data were first analysed using descriptive statistical methods. Measures of central tendency for continuous variables were displayed as means and their corresponding standard deviations or medians and their corresponding ranges’ lower and upper (Q25;Q75) values. The one way ANOVA and student’s t tests were used to compare continuously distributed variables whereas categorical variables were compared using the chi-squared and/or Fischer’s exact test accordingly. A descriptive time-series analysis with the C-statistic was used to analyse the yearly AEFI reporting trends as described by Tryon, 1982 [16]. The first 8 counts were used as the baseline data whilst the subsequent 12 counts were used to analyse whether there were any reporting trends [16]. The basic question was whether there was any demonstrable trend in yearly reporting since the establishment of AEFI reporting through the MCAZ. All statistical tests were done at the 5% significance level.

### Ethical considerations

Ethical exemption for the study was granted by the Medical Research Council of Zimbabwe (MRCZ Ref: MRCZ/E/207). The ethics review exemption was granted because AEFI reporting is a routine surveillance program which does not require informed consent and uses depersonalized, de-identified data.

## Results

### Demographic characteristics and quality of ICSR reports

**Fig. 1:**
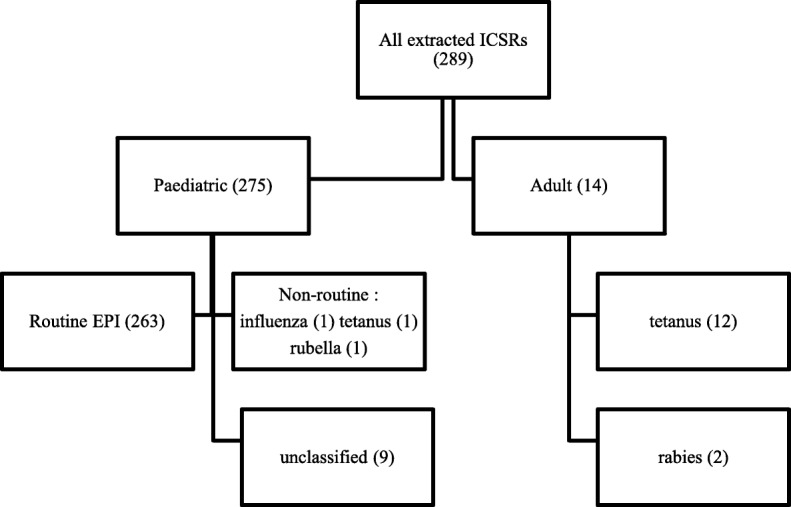
Schematic diagram showing the overall distribution of all ICSRs extracted from VigiBase®

During the period under review, two hundred and eighty nine (289) vaccine-related ICSRs were extracted from VigiBase®. This represents 10.4% of the 2777 medicine related ICSRs in the VigiBase® database from Zimbabwe on the date the data was extracted. A total of 17 ICSRs were excluded from further analysis because they had either a non-EPI or a non-paediatric vaccination indication as shown in Fig. 1. Therefore, a total of 272 ICSRs (263 routine EPI and 9 unclassified) were included for further analysis. Of the 272 ICSRs included in the analysis, 230 (84.6%) were reported by nurses, 35 (12.9%) were reported by physicians, 1(0.4%) report was submitted by a pharmacist whilst the remaining 6 (2.2%) were reported by unspecified healthcare professionals.

**Fig. 2:**
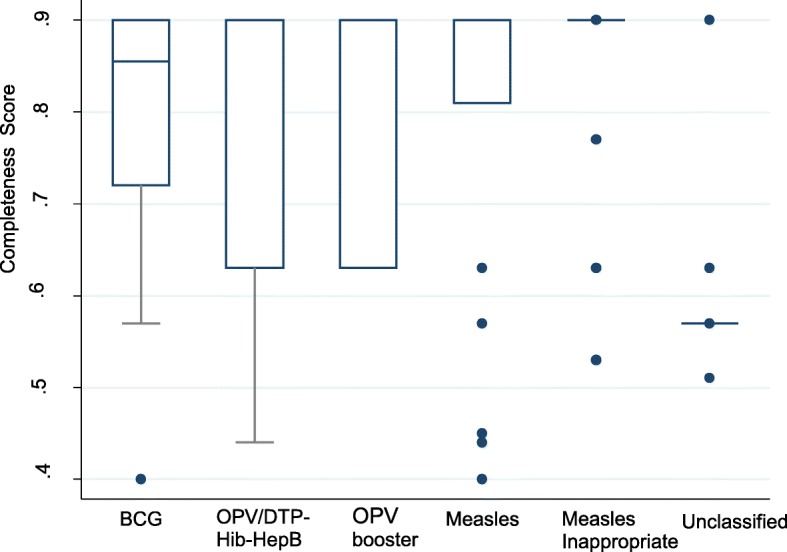
Box and Whisker plots ICSR reporting completeness scores for each vaccine/antigen

**Fig. 3:**
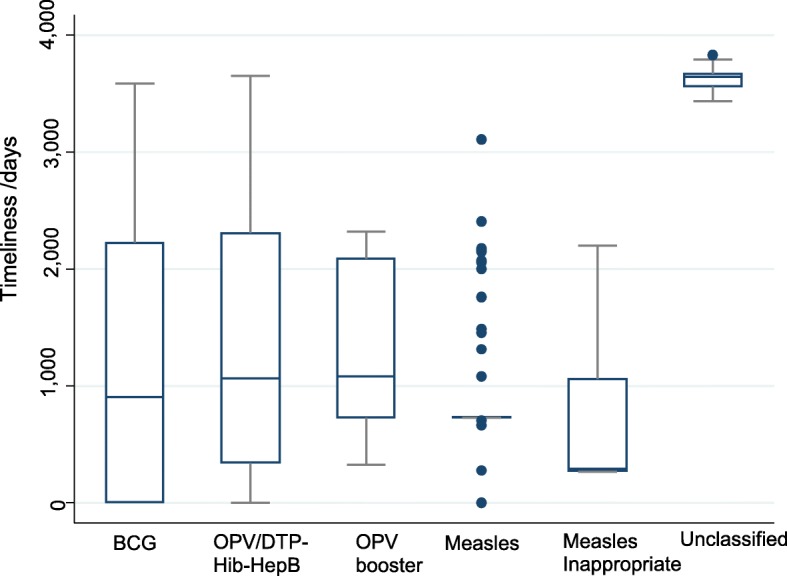
Box and Whisker plots ICSR reporting timeliness for each vaccine/antigen

Table 2 shows the demographic characteristics and the frequency of ICSRs across different antigens/vaccines. Overall, ICSRs were more frequently reported in males (55.4%) than females, although there was no association between ICSRs and gender (*p = 0.641*). The overall median age was 12 months (range 0–168) and the highest median age was observed in children vaccinated with measles (Median age = 72 months; range 9–168). The average completeness score was 0.81 ± 0.01, whilst the median score was 0.90 (IQR: 0.63; 0.90). ICSRs which originated from unclassified vaccines (0.61 ± 0.04) had the lowest completeness score. Figure 2 shows the completeness score ranges for ICSRs for the different vaccines. For ICSR entries with complete data, the average time from the onset of an AEFI to VigiFlow entry was 1205.94 ± 993.27 days whilst the median time was 734.00 days (IQR: 657.75; 2068.00 days). Figure 3 shows the timeliness of reporting for ICSRs for the different vaccines.

**Table 2:** Demographic characteristics and frequency of ICSRs per antigen or vaccine

Variable	Total ICSRs (*N* = 272) n (%)^a^	Vaccine						*P* value
		BCG (*N* = 16) n (%)^a^	Measles (*N* = 93) n(%)^a^	Measles inappropriate (*N* = 40) n (%)^a^	OPV/DTP booster (*N* = 7) n (%)^a^	OPV/DTP-Hib-HepB (*N* = 107) n (%)^a^	Unclassified (*N* = 9) n (%)^a^	
ICSR completeness Mean ± SE	0.81 ± 0.01	0.79 ± 0.04	0.82 ± 0.02	0.87 ± 0.01	0.82 ± 0.05	0.79 ± 0.02	0.61 ± 0.04	< 0.0001
Gender								
Male	150 (55.3)	7 (43.7)	55 (59.8)	22(55.0)	4 (57.1)	59 (55.1)	3(33.3)	0.641
Female	121(44.7)	9(56.3)	37(40.2)	18 (45.0)	3(42.9)	48 (44.9)	6(66.7)	
Male: female Ratio	1.24	0.78	1.49	1.22	1.33	1.23	0.50	
Age (months)								
Median	12	2.5	72	17	60	5	5	< 0.0001
Range	0–168	0–11	9–168	4–48	17–60	0–12	3–6	
Number of AEFIs^b^	387 (100.0)	20(5.2)	136(35.1)	60(15.5)	10(2.6)	152(39.3)	9(2.3)	< 0.0001
AEFI/ICSR ratio	1.42	1.25	1.46	1.50	1.43	1.42	1.00	
Severity of ICSR								
Serious	30(11.0)	2(12.5)	6(6.5)	7(17.5)	1 (14.3)	14(13.1)	0(0.0)	0.371
Not serious	242(89.0)	14(87.5)	87(93.5)	33(82.5)	6(85.7)	93(86.9)	9(100.0)	
Seriousness of ICSR								
Prolonged Hospitalisation	4(1.5)	0(0.0)	1(1.1)	0(0.0)	0(0.0)	3(2.8)	0(0.0)	0.051
Life Threatening	10(3.7)	0(0.0)	3(3.2)	4(10.0)	1(14.3)	2(1.9)	0(0.0)	0.156
Death	16(5.9)	2(12.5)	2(2.2)	3(7.5)	0(0.0)	9 (8.4)	0(0.0)	0.001

Key: ^a^column percentages; ^b^row percentages

### Vaccines

The majority of the 272 ICSRs could be grouped into the BCG, measles, OPV/DTP-Hib-HepB and OPV/DTP vaccination groupings. The proportions of ICSRs contributed by live attenuated vaccines (BCG and measles) and the combination doses with a mixture of inactivated, subunit and toxoid vaccines are shown in Table 2. The majority of ICSRs were reported in children who had received measles (*n* = 133; 48.9%), and OPV/DTP-Hib-HepB (*n* = 107; 39.3%) vaccines. However, some of the vaccinations were unclassified (*n* = 9; 2.3%). Measles was inappropriately administered outside the scheduled age in 40 children (14.7%).

### Outcomes and seriousness assessment

About 66.9% (259/387) of the reported AEFIs were recorded as either recovered or recovering with the remainder recorded as either an unknown outcome or death. The prevalence of serious events were calculated using the total number of ICSRs and the number of ICSRs reported per vaccine as the denominator. Of the total 272 reported ICSRs, 30 (11.0%) were recorded as serious events. Of the serious events, 16 (5.9%) were deaths, 10(3.7%) were life threatening, and 4(1.5%) were prolonged hospitalizations. Serious events occurred with higher frequencies in children who received OPV/DTP-Hib-HepB (*n* = 14; 13.1%) and measles inappropriately (*n* = 7; 17.5%). Out of the seven deaths that occurred in neonates, five were observed in children who had inappropriate (wrong age) administration of OPV/DTP-Hib-HepB. BCG (*n* = 2; 12.5%), OPV/DTP-Hib-HepB (*n* = 9; 8.4%), and measles administered inappropriately (*n* = 3; 7.5%) were associated with higher frequencies of deaths compared to other vaccines (*p* = 0.001). However, no causality information was recorded to assess the relatedness of the vaccine/antigens to the deaths.

### Adverse event distribution

There were more systemic AEFIs compared to local AEFIs reported during the period under review. Systemic events were more frequently reported with doses containing the measles antigen (*n* = 190; 49.1%) while local events were associated with the multiple antigen OPV/DTP-Hib-HepB vaccine (*n* = 62; 16.0%). The most common systemic AEFIs were pyrexia (*n* = 61; 15.8%) and rash (*n* = 49; 12.7%). The most frequently reported local AEFI was the injection site abscess (*n* = 75; 19.4%). The multiple antigen OPV/DTP-Hib-HepB vaccine was associated with higher rates of injection site abscess (*n* = 57; 37.5%), pyrexia (*n* = 27; 17.8%), diarrhoea (*n* = 15; 9.9%), vomiting (*n* = 12; 7.9%), and seizures (*n* = 6; 3.9%). The measles antigen was associated with higher rates of rash (*n* = 44; 22.4%), ocular disorders (*n* = 17; 8.7%), pyrexia (*n* = 26; 13.3%), urticaria (*n* = 22; 11.2%), diarrhoea (*n* = 8; 4.1%), vomiting (*n* = 12; 6.1%), hypersensitivity reactions (*n* = 4; 2.0%). Table 3 shows the distribution of AEFIs across different antigens/vaccines.

**Table 3:** Distribution of AEFIs across antigens/vaccines

Variable	Total number of AEFIs (*N* = 387) n (%)^a^	Vaccine						*P* value
		BCG (*N* = 20) n (%)^a^	Measles (*N* = 136) n (%)^a^	Measles inappropriate(*N* = 60) n (%)^a^	OPV/DTP booster (*N* = 10) n (%)^a^	OPV/DTP-Hib-HepB (*N* = 152) n (%)^a^	Unclassified (*N* = 9) n (%)^a^	
Type of AEFI								
Systemic	301(77.8)	13(65.0)	130(95.6)	60 (100.0)	6 (60.0)	90(59.2)	2(22.2)	< 0.0001
Local	86(22.2)	7(35.0)	6(4.4)	0(0.0)	4(40.0)	62(40.8)	7(77.8)	
Systemic AEFIs								
Cough	5(1.3)	0	3	1	0	1	0	0.146
Diarrhoea	23(5.9)	0	3	5	0	15	0	< 0.0001
Dyspnoea	4(1.0)	0	1	0	0	3	0	0.051
Hypersensitivity	4(1.0)	0	4	0	0	0	0	0.001
Occular Disorders	17(4.4)	0	14	3	0	0	0	< 0.0001
Paralysis	4(1.0)	0	1	1	1	1	0	0.841
Pyrexia	61(15.8)	4	13	13	3	27	1	< 0.0001
Seizure	12(3.1)	0	2	4	0	6	0	0.007
Vomiting	25(6.5)	0	4	8	0	12	1	< 0.0001
Pruritus	11(2.8)	0	9	2	0	0	0	< 0.0001
Rash	49(12.7)	1	33	11	1	3	0	< 0.0001
Urticaria	23(5.9)	1	19	3	0	0	0	< 0.0001
Other	63(16.3)	7	24	9	1	22	0	
Local AEFIs								
Injection site Abscess	75 (19.4)	7	3	0	3	57	5	< 0.0001
Injection site Swelling	5(1.3)	0	0	0	1	3	1	0.146
Other	6(1.6)	0	3	0	0	2	1	0.156

Key: ^a^column percentages

### Reporting trends

**Fig. 4:**
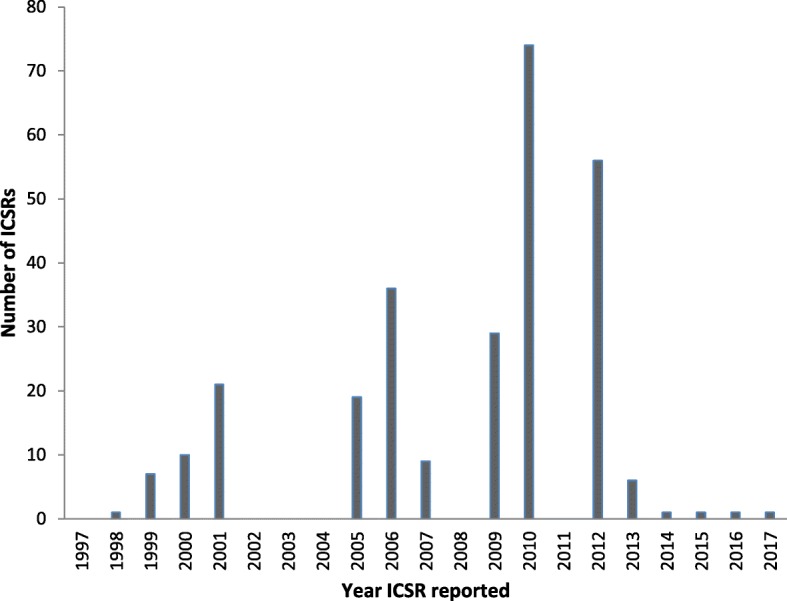
Annual ICSR reporting trends for the period 1997 to 2017

Table 4 shows the AEFI reporting rates for the individual vaccines. The overall AEFI reporting rate was 0.58 per 100,000 vaccine doses and the AEFI/ICSR ratio was 1.42. Measles had the highest AEFI reporting rate of 2.11 per 100,000 followed by OPV/DTP-Hib-HepB at 0.54 per 100,000 vaccine doses. The highest AEFI/ICSR ratio was observed when measles was administered inappropriately (1.50) and the lowest ratio was observed with BCG vaccine (1.25). From 1997 to 2010, there was a general increase in the number of ICSRs. The highest annual count of ICSRs was observed in 2010 (*n* = 74; 27.2%). The annual counts of the ICSRs reported during the period 1997 to 2017 are shown in Fig. 4. The annual AEFI reporting ratio ranged between 0 and 30.2 per 100,000 surviving infants. Table 5 shows the annual number of ICSRs, AEFIs, and AEFI reporting ratios from 1997 to 2017. There was a significant increase in the reporting of ICSRs during the baseline and the reporting periods (*p < 0.05)*.

**Table 4:** Number and rates of AEFIs per vaccine/antigen from 1997 to 2017

Vaccine/Vaccine combination	Number of doses per birth cohort^a^	Number of AEFIs	Number of vaccine doses (million)	Rate per 100,000 vaccine doses
BCG	1	20	9.3	0.21
Measles	1	196^b^	9.3	2.11
OPV/DTP-Hib-HepB	3	152	27.9	0.54
OPV/DTP booster	2	10	18.6	0.05
Total		378^c^	65.1	0.58^d^

Key: ^a^Number of doses per birth cohort is based on the Zimbabwe Expanded Immunisation Programme schedule used prior to 2012. ^b^Includes all AEFIs reported with measles regardless of the age at which the vaccine was administered. ^c^Excludes AEFIs reported for unclassified vaccines. ^d^Average reporting rate per 100,000 vaccine doses

**Table 5:** Number of ICSRs and annual AEFI ratios from 1997 to 2017

Reporting year	Number of ICSRs	Number of AEFIs	Number of surviving infants	Annual AEFI ratio per 100,000 surviving infants
1997	0	0	426,000	0
1998	1	1	427,000	0.2
1999	7	12	433,000	2.8
2000	10	12	364,000	3.3
2001	21	28	362,000	7.7
2002	0	0	361,000	0
2003	0	0	360,000	0
2004	0	0	360,000	0
2005	19	23	351,000	6.5
2006	36	53	352,000	15.5
2007	9	10	354,000	2.8
2008	0	0	356,000	0
2009	29	44	359,000	12.3
2010	74	107	354,000	30.2
2011	0	0	358,000	0
2012	56	84	501,000	16.8
2013	6	7	507,000	1.4
2014	1	2	511,000	0.4
2015	1	1	512,000	0.2
2016	1	1	512,000	0.2
2017	1	1	515,000	0.2
Total^a^	271^a^	385^b^		

^a^Excludes one ICSR which had missing information on the year the report was made

^b^Excludes two AEFIs from the ICSR with missing information on the year the report was made

## Discussion

The main purpose of this descriptive study was to provide a detailed analysis of the characteristics, quality and reporting trends of AEFIs captured from the Zimbabwe Expanded Immunisation Programme (ZEPI) as recommended by the WHO’s Global Manual in the surveillance of AEFI [1]. Data extracted from the WHO’s global pharmacovigilance database, VigiBase®, for Zimbabwe for the period 1997 to 2017 was used as it represented the entire AEFI dataset submitted by the national pharmacovigilance centre. We found an overall AEFI reporting rate of 0.58 per 100,000 vaccine doses and the annual AEFI reporting ratio ranged between 0 and 30.2 AEFI reports per 100,000 surviving infants. There was a steady increase in the AEFI reporting trends over time. The peak in AEFI reporting observed in 2009 could have been due to enhanced vaccine safety awareness associated with the pandemic H1N1 influenza virus vaccination campaign during this period as also observed by Hu et al. in Zhenjiang, China [17]. The decrease in the reporting rates observed from 2014 could be due to pending collection and uploading of the ICSRs into VigiBase® by the national pharmacovigilance centre. The widely varying annual AEFI reporting ratio resultant from the varying AEFI reporting trends indicates that Zimbabwe’s vaccine safety system is still developing. A functional AEFI reporting system should have a minimal annual reporting ratio of 10 AEFI reports per 100,000 surviving infants as indicated in the WHO’s Global Vaccine Action Plan for vaccine safety monitoring [14].

The observed 11.0% of AEFIs classified as serious is consistent with findings from passive surveillance systems observed in Australia (11%), the United States (14.2%) and Germany (19%), but it differs markedly from the findings in Zhejiang province, China (1%) and Croatia (3%) [17–22]. These differences may be due to the inherent differences in the reporting practices of the different countries [17]. Similar to the observations made by Zhou et al. in China, pyrexia (15.8%) was the most frequently reported AEFI and this commonly followed receipt of a dose of pertussis-containing vaccines (DTP) than for other vaccines [23]. However, in the absence of causality assessment, we are not able to make similar conclusions concerning neonatal deaths which occurred near the dates of vaccination. In a country with a high infant and childhood mortality, these could be coincidental deaths. The deaths could also be attributed to other conditions associated with high infant and childhood mortality and this necessitates robust investigations and causality assessment to rule out other causes [24]. The high overall and vaccine specific male to female reporting ratios are similar to the findings in Australia in children below 12 months of age for AEFIs and below 5 years of age for other medicines [21, 25]. However, this observation differs from the findings in a study by Harris et al. which concluded that the relative reporting rate for AEFIs tends to be equal in children below 11 years of age [26]. The reasons for the AEFI preponderance in infant males are not clear.

The administration of multiple antigens or vaccines tends to produce a widening and lowering of the ICSR completeness score ranges. However, the AEFI completeness scores were markedly high compared to the global average of 0.41 observed within VigiBase® [27, 28]. The median time interval between the date of onset of an ADR and the date of reporting to VigiBase® of 734.00 days (≈ 2.01 years) is comparable to the 2.40 years observed in a review of global ICSR data [29, 30]. However, the lack of follow-up data prevents a full assessment of the quality of the AEFI data. Furthermore, the zero annual reporting observed for some of the years under review suggests under-reporting as vaccines are regularly and consistently administered by the national EPI programme. Under-reporting in Zimbabwe is also highlighted by the rather low overall AEFI reporting ratio of 0.58 AEFIs per 100,000 doses. Although, this AEFI reporting rate is comparable to the 2.7 AEFIs per 100,000 doses observed in Switzerland, it [31] is lower than the Australian overall annual AEFI reporting ratio of 14.6 AEFIs per 100,000 doses in children under the age of 7 years for all scheduled vaccines [32] and the 12.4 AEFIs per 100,000 doses reported in the Spanish passive AEFI surveillance system [33]. The underdevelopment of the passive AEFI surveillance system in Zimbabwe may explain the low AEFI reporting rates compared to other AEFI surveillance systems. Furthermore, inadequate healthcare worker training on AEFI surveillance, limited coordination of AEFI surveillance activities, insufficient reporting resources and competing work priorities may also adversely affect the AEFI reporting trends in Zimbabwe [14, 34]. It should also be noted that all the AEFI reporting rates observed in Australia, Spain, Switzerland, and Zimbabwe are way lower than the global average rate of 549 AEFIs per 100,000 surviving infants reported in 2015. This global average was largely driven by the markedly high Eastern Mediterranean region’s AEFI report submissions [14]. The observed AEFI reporting variations may be explained by regional differences in immunization program funding, population demographics, and AEFI reporting requirements and case definitions [35]. In addition, passive AEFI surveillance rates can be influenced by active vaccine surveillance programs and enhanced vaccine safety awareness campaigns.

Several strategies can be employed to strengthen the AEFI surveillance system in Zimbabwe. Improving healthcare professionals’ awareness of vaccine safety reporting and providing appropriate and timely feedback to reporters of AEFIs may increase the quality and the numbers of reported AEFIs [36]. In addition, complementary increases in internal funding and resources such as fully functional, identifiable sentinel AEFI reporting sites, the linkage of AEFI data with immunisation registers, and the use of national vaccine safety experts may also enhance patient follow-up, AEFI investigations and the timeliness and completeness of ICSRs [36]. Furthermore, the use of participant-centred active surveillance of AEFIs can be utilised instead of passive surveillance as it is sustainable, relatively affordable and provides timely signal detection opportunities especially for post-marketing vaccine pharmacovigilance [37]. Given the shift towards vaccine development for tropical and other health challenges in resource limited settings, active, participant-centred safety surveillance may offer a unique solution to provide complete, timely and useful AEFI data. Furthermore, pharmacovigilance information documented within the UMC VigiBase® system is important for monitoring vaccine safety signals [38]. Therefore, the utility of the VigiBase® data can be increased by the addition of causality assessments and reporter assessed cause-specific categorisation of AEFIs during data entry. This will provide sufficient information for the comprehensive analysis AEFI safety data. In addition, cause-specific categorisation will enable the differentiation of coincidental events from vaccine reactions especially for events such as death whose occurrence and miscommunication with the community may disrupt the EPI programme [24].

This study has several limitations that should be borne in mind when interpreting these findings. First, the passive AEFI surveillance system has inherent limitations which include under-reporting of AEFI reports, inadequate training of healthcare workers in basic AEFI surveillance, variability in the accuracy and completeness of AEFI reports, and inconsistency in the application of case definitions for reportable AEFIs. Second, the AEFI rates were calculated using the birth cohort as an estimate of the number of children who were vaccinated. Therefore, the unavailability of the accurate denominator data (number of doses administered to patients) makes the AEFI rates less accurate. Although using the population of a birth cohort can lead to less accurate AEFI rates, this approach can serve as a good indicator of the performance of the AEFI surveillance system over time. Third, the lack of data on causality assessments in VigiBase® makes it difficult to make any conclusions regarding potential causal relationships between vaccines and specific AEFIs. Furthermore, passive AEFI surveillance systems have an inherent limitation that the observed temporal relationships between immunizations and AEFI cases do not necessarily mean that they are causal. Fourth, this study is based on AEFIs submitted to the UMC VigiBase® database after verification for validity by the Medicines Control Authority of Zimbabwe. AEFIs with incomplete or inconsistent information submitted to MCAZ are returned to the healthcare institutions for additional data. This censoring of ICSR data has the potential of reducing the number of reported ICSRs and inflating the quality of analysed ICSRs. In addition, the de-duplication algorithm in VigiBase®, VigiMatch®, further censors the available reports with possible misrepresentation of the reporting quality and the number of ICSRs [39]. This presents challenges to the validity of analyses done using the data from VigiBase® by most developing vaccine safety reporting systems [39]. Therefore, the results of this study may underestimate the occurrence of AEFIs. Lastly, the limited follow-up data, especially for serious AEFIs, reduced our ability to fully assess the performance of the reporting system and the efficiency of assessing the circumstances around the serious adverse events. In addition, the lack of identifiable reporting health institutions reduced our ability to analyse whether there were any reporting differences between sites. Despite these limitations, this study clearly shows the performance of AEFI surveillance system in Zimbabwe.

## Conclusions

Most of the ICSRs were associated with measles and OPV/DTP-Hib-HepB vaccines while the majority of the AEFIs were systemic events. The overall AEFI reporting rate was low suggesting under-reporting of AEFIs within the vaccine safety surveillance system. Zimbabwe’s vaccine safety surveillance system is still developing and is not yet fully functional. However, the current system provides a reference point for the monitoring of the ongoing AEFI reporting trends and characteristics. This interim analysis provides areas of the vaccine safety surveillance that need improvement such as the recording of causality assessments, cause-specific categories of AEFIs and the need to indicate the reporting centres to derive maximum benefit from future analyses. In addition, adequate healthcare worker training on AEFI surveillance, improved coordination of AEFI surveillance activities, and improved reporting resources will strengthen the vaccine safety surveillance system.

## Data Availability

Data from the WHO Collaborating Centre for International Drug Monitoring were used. The datasets used and/or analysed during the current study are available from the corresponding author on reasonable request. However, the authors will abide to the restrictions imposed by WHO policy and the Uppsala Monitoring Centre (UMC) guidelines on sharing the datasets.

## Abbreviations

AEFI: Adverse Events Following Immunisation
CIOMS: Council for International Organizations of Medical Sciences
EPI: Expanded Programme on Immunisation
GAVI: Global Alliance for Vaccines and Immunisation
GVAP: Global Vaccine Action Plan
ICSR: Individual Case Safety Reports
MCAZ: Medicines Control Authority of Zimbabwe
PIDM: Programme for International Drug Monitoring
WHO: World Health Organization
